# Supplementary material for: Inflammatory factors and risk of lung adenocarcinoma: a Mendelian randomization study mediated by blood metabolites
Source: Front Endocrinol (Lausanne). 2024 Aug 27;15:1446863. doi: 10.3389/fendo.2024.1446863 (PMC11384989; doi:10.3389/fendo.2024.1446863)
Supplement: Supplementary file 1 [file DataSheet1.zip › supplementary files/S2/GCST90200586/forest.pdf]

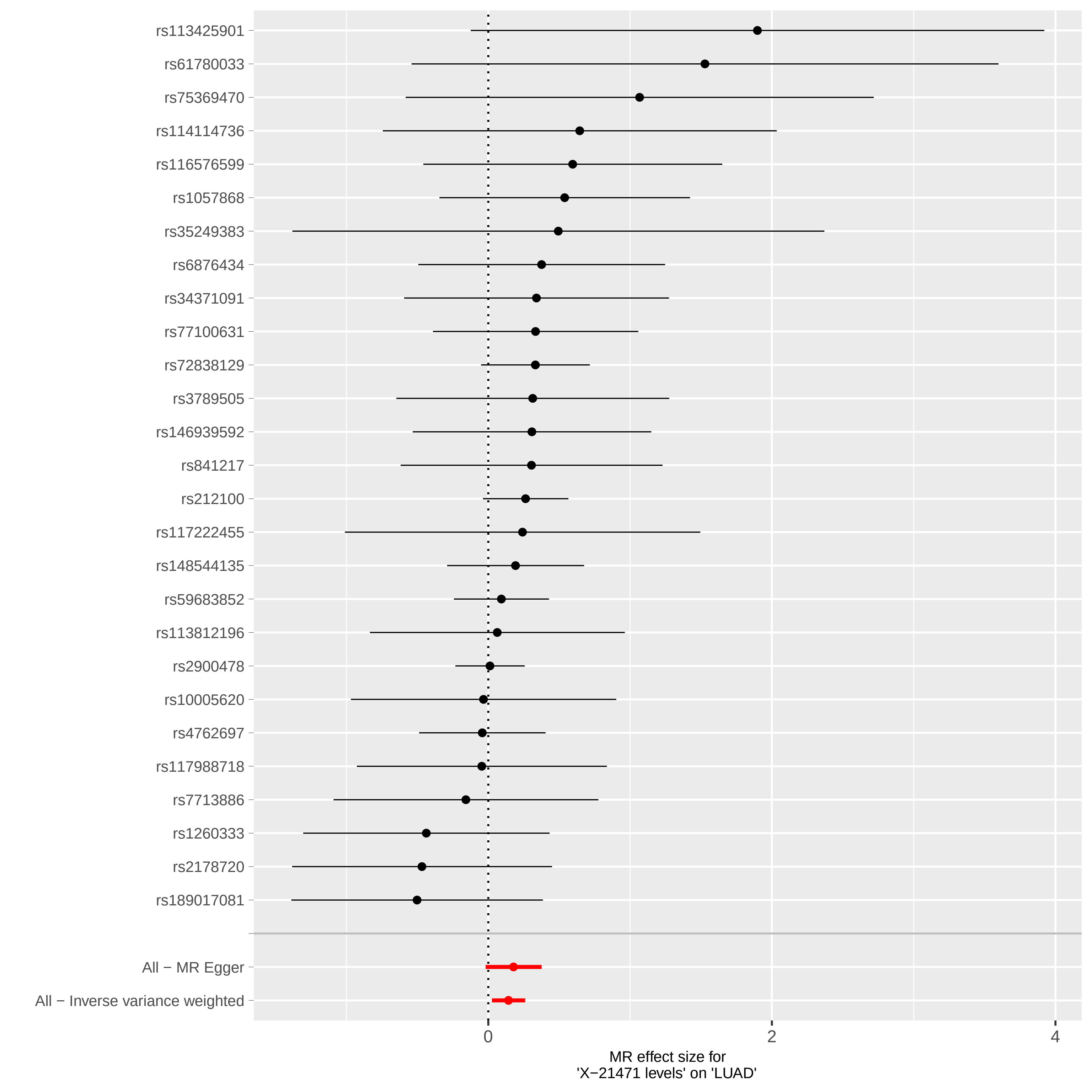
rs113425901
rs61780033
rs75369470
rs114114736
rs116576599
rs1057868
rs35249383
rs6876434
rs34371091
rs77100631
rs72838129
rs3789505
rs146939592
rs841217
rs212100
rs117222455
rs148544135
rs59683852
rs113812196
rs2900478
rs10005620
rs4762697
rs117988718
rs7713886
rs1260333
rs2178720
rs189017081
All − MR Egger
All − Inverse variance weighted
0
2
4
MR effect size for
'X−21471 levels' on 'LUAD'
